# De novo gastric cancer developing after liver transplantation from deceased donor for biliary atresia: a case report

**DOI:** 10.1186/s40792-021-01210-x

**Published:** 2021-05-18

**Authors:** Naruki Higashidate, Suguru Fukahori, Shinji Ishii, Nobuyuki Saikusa, Naoki Hashizume, Yoshinori Koga, Daisuke Masui, Saki Sakamoto, Shiori Tsuruhisa, Hirotomo Nakahara, Yoshiaki Tanaka, Masaru Fukahori, Keisuke Miwa, Yoshiki Naito, Minoru Yagi

**Affiliations:** 1 Department of Pediatric Surgery, Kurume University School of Medicine, 67 Asahi-machi, Kurume, Fukuoka 830-0011 Japan; 2 Division of Medical Safety Management, Kurume University Hospital, Kurume, Japan; 3 Multidisciplinary Cancer Treatment Center, Kurume University Hospital, Kurume, Japan; 4 Department of Diagnostic Pathology, Kurume University Hospital, Kurume, Japan

**Keywords:** Biliary atresia, Liver transplantation, De novo malignancy, Gastric cancer

## Abstract

**Background:**

Apart from Kasai’s procedure, liver transplantation (LTx) has dramatically improved the outcome of children with biliary atresia (BA). However, de novo malignancy has been reported to be one of the major causes of late mortality after LTx among adults. We report a rare case of de novo gastric cancer developing after LTx for BA received during childhood.

**Case presentation:**

A 21-year-old male patient who had undergone LTx for BA at age 2 years occasionally visited our outpatient clinic due to symptoms of epigastric pain and dysphagia. Endoscopic examination and computed tomography revealed advanced gastric cancer at the gastroesophageal junction with multiple liver metastases. Despite systemic chemotherapy, the disease progressed, resulting in patient’s death 2 years after the diagnosis.

**Conclusions:**

De novo malignancy in the absence of post-transplant lymphoproliferative disease is rare in pediatric patients who received LTx. To the best of our knowledge, no report has been available on the development of gastric cancer after LTx for BA during childhood. Primary physicians should therefore establish a follow-up plan for patients receiving LTx for BA considering the potential for the development of de novo malignancy, including gastric cancer, despite its rarity.

## Background

Biliary atresia (BA) is the most devastating neonatal and early infancy liver disease. Nonetheless, Kasai’s procedure and liver transplantation (LTx) have dramatically improved outcomes among children with BA, with the Japanese Biliary Atresia Registry reporting the current 20-year overall survival rate to be 89% [1]. Meanwhile, reports have shown the development of de novo malignancy to be one of the major causes of late mortality after LTx among the adult population. A recent national survey in Japan had identified post-transplant lymphoproliferative disease (PTLD), lung cancer, and colon cancer as the top three conditions associated with de novo malignancies arising after LTx [2]. Among the pediatric population, PTLD is the most common cause of de novo malignancy following organ transplantation [3, 4]. Unlike adult patients, de novo solid malignant tumors are rare in pediatric patients receiving organ transplantation, with only two cases involving liver sarcoma and hepatocellular carcinoma having been reported [5, 6]. Meanwhile, no report has been available regarding the development of gastric cancer after LTx for BA.

We herein report a rare case of de novo gastric cancer developing after LTx for BA during childhood.

## Case presentation

**Fig. 1:**
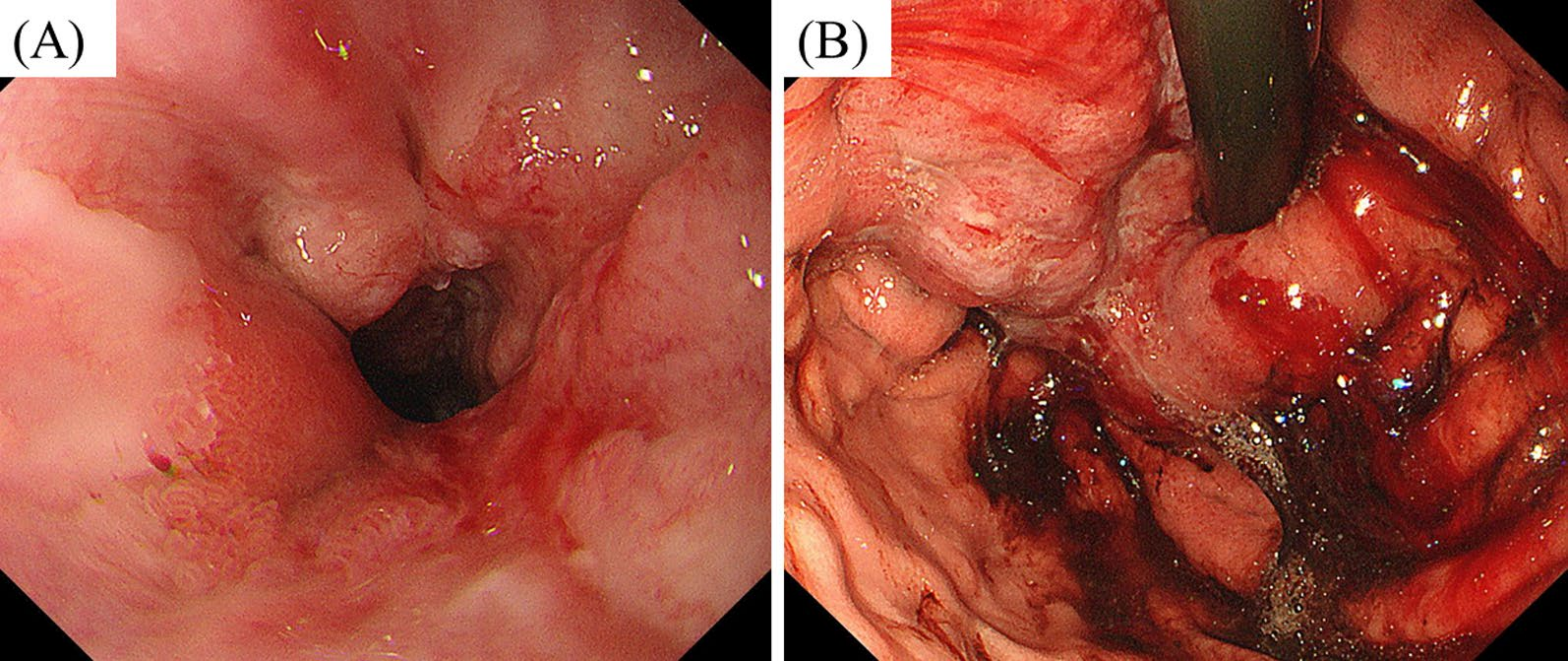
**A** The tumor occupying the entire circumference of the gastroesophageal junction. **B** The tumor was associated with hemorrhage and ulcer formation in the central area

**Fig. 2:**
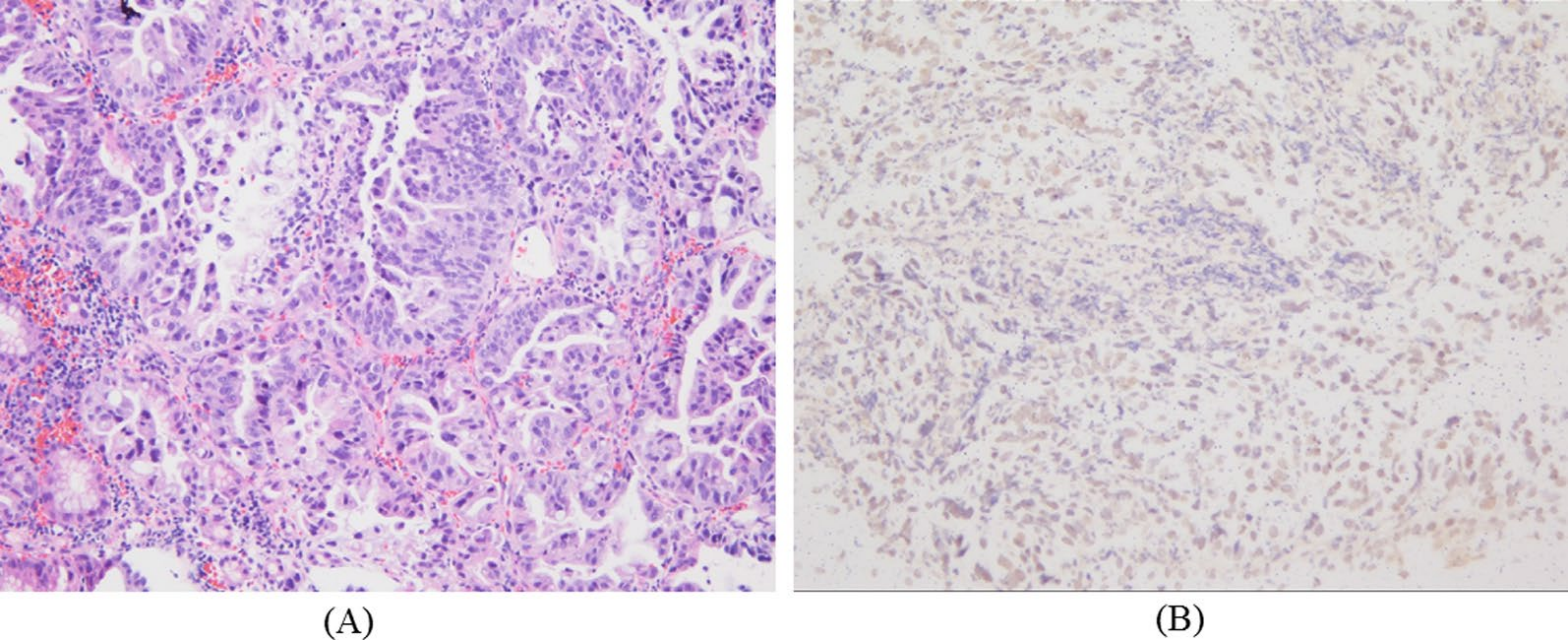
**A** Histopathological examination revealing moderately differentiated adenocarcinoma (hematoxylin and eosin staining, × 100). **B** Negative Epstein–Barr virus-encoded small RNA in situ hybridization (EBIS staining, × 100)

**Fig. 3:**
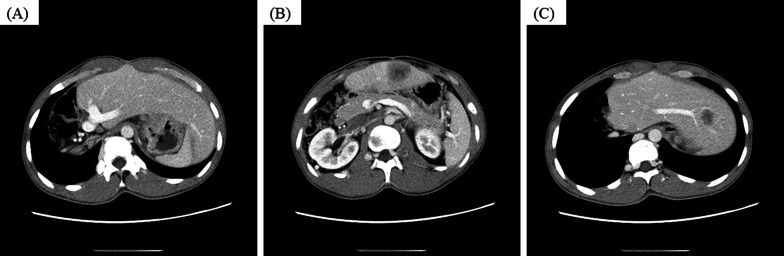
**A** Contrast-enhanced computed tomography revealing tumor invasion beyond the serosal layer. **B**, **C** Multiple metastatic lesions in the transplanted liver

A 21-year-old male patient occasionally visited our outpatient clinic due to symptoms of epigastric pain and dysphagia. His past medical history included BA during infancy for which he had undergone Kasai’s procedure when 5 months old and LTx from a deceased donor at age 2 years due to advanced liver failure. Patient family history included the death of his father due to tongue cancer. The patient had received tacrolimus as immunosuppressive treatment and underwent routine blood tests at our outpatient clinic. During the patient’s adult years, his liver function was almost within the normal range, with trough levels of tacrolimus ranging from 1.0 to 1.5 ng/mL at our outpatient clinic. Although he had slight mental retardation, he was able to achieve a normal school life. At the age of 20, he often experienced general fatigue and appetite loss, which were improved by simple rehydration. Upper gastrointestinal endoscopic examination revealed the presence of a type Ш tumorous lesion at the gastroesophageal junction (Fig. 1A, B). No obvious abnormal findings were noted in the non-cancerous area of the stomach. Histological evaluation of the biopsied specimen demonstrated moderately differentiated adenocarcinoma (Fig. 2A). Immunohistopathological examination of the tumor cells revealed that he was positive for human epidermal growth factor type 2 (HER-2) but negative for Epstein–Barr virus (EBV)-encoded small RNA in situ hybridization (EBER-ISH) (Fig. 2B). Contrast-enhanced computed tomography revealed tumor invasion beyond the serosal layer of the stomach and extensive areas of lymph node enlargement, including the para-aortic region. Moreover, multiple metastatic lesions were detected in the liver (Fig. 3A–C). Based on the aforementioned findings, the patient was diagnosed with stage IV advanced gastric cancer. Systemic chemotherapy comprising trastuzumab, capecitabine, and oxaliplatin was selected according to the therapeutic guidelines for gastric cancer in Japan. After initiating first-line chemotherapy, his symptoms improved, with an apparent shrinking of the tumors on diagnostic imaging. However, he was transferred to second-line therapy with nab-paclitaxel plus ramucirumab due to adverse effects to capecitabine, such as severe toe paronychia and numbness in the extremities. Unfortunately, the patient developed resistance to chemotherapy, and eventually succumbed to his disease 2 years after the diagnosis.

## Discussion

The recent advances in surgical maneuvers and perioperative care, as well as optimized use of immunosuppressive agents, have improved the clinical outcomes of LTx. Among the adult population; however, de novo malignancy has been reported as one of the major causes of late mortality after LTx. Non-Hodgkin lymphoma, skin cancer, and Kaposi’s sarcoma have been reported as the most common malignancies developing after LTx [7]. Several major risk factors have been associated with the development of de novo malignancy after LTx, including advanced age, alcohol consumption, smoking, oncogenic viruses, long-term use of immunosuppressive drugs, and others [8]. The impaired anti-carcinogenesis actions of T lymphocytes, macrophages, and natural killer cells for immune surveillance, delaying tumor progression, and preventing angiogenesis, vascular invasion, and metastasis have been proposed as the mechanisms by which tumorgenesis develops in patients with immunosuppressive therapy [9]. *Helicobacter pylori* and EBV infections have also been known as risk factors for gastric cancer in patients with LTx, with the eradication of these microorganisms possibly being necessary for reducing the risk of de novo gastric cancer in transplant recipients [10]. However, the present patient exhibited no obvious specific findings to suspect *Helicobacter pylori* infection, with his tumor specimen being immunopathologically negative for EBV. A family history of tongue cancer in his father and the use of tacrolimus had been considered to contribute toward the development of de novo gastric cancer in the present case.

In particular, de novo gastric cancer developing after LTx is rare in Western countries. A study in South Korea reported that de novo malignancy occurring after LTx in adults most commonly develops in the stomach, with an incidence rate of 0.64%—a figure higher than that in Western countries [11]. Recently, a national survey in Japan had published findings regarding de novo malignancies developing after solid organ transplantation in adult patients [2], with post-LTx malignancy most frequently involving sites of being PTLD (18.3%), lung cancer (12.9%), colon cancer (11.8%), and stomach cancer (10.2%). Thus, Asian countries including Japan, have higher incidence rates of de novo gastric cancer after LTx compared to Western countries, which could be attributed to differences in patient characteristics.

Meanwhile, PTLD has been reported to be the most common and important post-LTx-associated malignancy among pediatric patients, with prevalence rates ranging from 2–27% [3, 4], accounting for 80% of all de novo malignancies [12]. The development of PTLD in pediatric patients is usually secondary to EBV infection and immunosuppression [13]. However, de novo solid malignant tumors are generally rare in pediatric patients who receive LTx. A previous survey from Turkey on de novo malignancy in pediatric patients receiving LTx for BA reported that 13/206 (6.3%) pediatric patients with LTx were diagnosed with de novo malignancies, including 7 patients with BA [6]. Among them, one case of liver sarcoma had been included as a de novo solid malignant tumor. Moreover, only one case of solid organ malignancy after LTx for BA had been reported. Accordingly, this case involved de novo hepatocellular carcinoma occurring 14 years after LTx for BA, which developed from progressive liver fibrosis in the absence of underlying viral infections [5]. To the best of our knowledge, no report has been available on patients developing gastric cancer after LTx for BA.

As described earlier, prolonged immunosuppressive therapy may increase the risk of developing de novo malignancy, with one study identifying tacrolimus as an independent risk factor for post-transplant malignancy developing in patients who had undergone renal transplantation [14]. Indeed, studies have showed that tacrolimus was associated with the risk of solid organ tumors, not limited to gastric cancer [10]. Given that the present case had been receiving tacrolimus for approximately 19 years after LTx, such long-term use of tacrolimus might have contributed to the development of gastric cancer. Although careful selection of immunosuppressive drugs could be a preventive measure against post-transplant malignancy, no replacement for tacrolimus is available in the current clinical settings throughout Japan. A previous analysis of adult LTx recipients who had received tacrolimus revealed a dose–response relationship between tacrolimus and the occurrence rate of solid cancer [15]. However, we could not find any study indicating a safe tacrolimus trough for the prevention of carcinogenesis. Therefore, careful monitoring of tacrolimus levels to avoid excessive immunosuppression is imperative.

To reduce cancer-related mortality and morbidity, early detection and treatment of post-transplant and ordinary malignancies are crucial. Generally, patients who develop de novo malignancies after organ transplantation tend to present with advanced disease stage upon diagnosis, resulting in poor prognosis [16]. We did not consider providing any additional examinations in the present case apart from the routine blood tests (e.g., for malignancy screening) until after the patient started complaining about his symptoms. Had earlier upper gastrointestinal endoscopy been scheduled, his gastric cancer might have been detected and treated at an earlier stage. However, to date, no protocol for cancer screening after organ transplantation has yet been established in Japan, even for adult patients. Considering the expected increase in the number of long-term survivors following LTx for BA, strategies for long-term follow-up should be established to enable the timely detection and treatment of de novo malignancies. We recommend upper and lower gastrointestinal endoscopic examination at least once a year among organ transplantation recipients considering the higher incidence rates of de novo gastric and colorectal cancer in Japan compared to Western countries. Moreover, pediatric patients with organ transplantation should receive careful surveillance for solid organ malignancies.

## Conclusions

We herein detail our experience with a rare case of de novo gastric cancer developing after LTx received during childhood for BA. Primary physicians should therefore schedule follow-up plans considering the potential for patients receiving LTx for BA to develop de novo malignancy, including gastric cancer, despite its rarity.

## Data Availability

The datasets supporting the conclusions of this article are included within the article.

## Abbreviations

LTx: Liver transplantation
BA: Biliary atresia
PTLD: Post-transplant lymphoproliferative disease
HER-2: Human epidermal growth factor type 2
EBV: Epstein–Barr virus
EBER-ISH: Epstein–Barr virus-encoded small RNA in situ hybridization
